# Implications of Fine-Grained Habitat Fragmentation and Road Mortality for Jaguar Conservation in the Atlantic Forest, Brazil

**DOI:** 10.1371/journal.pone.0167372

**Published:** 2016-12-14

**Authors:** Laury Cullen, Jessica C. Stanton, Fernando Lima, Alexandre Uezu, Miriam L. L. Perilli, H. Reşit Akçakaya

**Affiliations:** 1 IPÊ –Instituto de Pesquisas Ecológicas, Nazaré Paulista, São Paulo, Brazil; 2 Department of Ecology and Evolution, Stony Brook University, Stony Brook, New York, United States of America; 3 Programa de Pós-graduação em Ecologia e Biodiversidade, Instituto de Biociências, Universidade Estadual Paulista–UNESP, Rio Claro, São Paulo, Brazil; 4 Instituto para a Conservação dos Carnívoros Neotropicais–Pró-Carnívoros, Atibaia, São Paulo, Brazil; 5 Programa de Pós-graduação em Ecologia, Departamento de Biologia Geral, Universidade Federal de Viçosa—UFV, Viçosa, Minas Gerais, Brazil; Australian National University, AUSTRALIA

## Abstract

Jaguar (*Panthera onca*) populations in the Upper Paraná River, in the Brazilian Atlantic Forest region, live in a landscape that includes highly fragmented areas as well as relatively intact ones. We developed a model of jaguar habitat suitability in this region, and based on this habitat model, we developed a spatially structured metapopulation model of the jaguar populations in this area to analyze their viability, the potential impact of road mortality on the populations' persistence, and the interaction between road mortality and habitat fragmentation. In more highly fragmented populations, density of jaguars per unit area is lower and density of roads per jaguar is higher. The populations with the most fragmented habitat were predicted to have much lower persistence in the next 100 years when the model included no dispersal, indicating that the persistence of these populations are dependent to a large extent on dispersal from other populations. This, in turn, indicates that the interaction between road mortality and habitat fragmentation may lead to source-sink dynamics, whereby populations with highly fragmented habitat are maintained only by dispersal from populations with less fragmented habitat. This study demonstrates the utility of linking habitat and demographic models in assessing impacts on species living in fragmented landscapes.

## Introduction

Loss of natural vegetation cover often leads to a fragmented distribution of habitat. How this habitat fragmentation affects species depends on the spatial scale and pattern of the fragmentation in relation to how the species uses the landscape [1]. One level of fragmentation may result in an environment perceived as "coarse-grained" by a small species with limited home range and dispersal [2]. In this case, each fragment may be large enough to contain a subpopulation, or a part of it. Thus, fragmentation divides a large population into multiple subpopulations, with a total carrying capacity less than that of the original single population. The same level of fragmentation may result in an environment perceived as "fine-grained" by a larger, more mobile species. In this case each population (even each territory) may extend to multiple fragments [3,4]. The population-level effects in this case are likely to be more complex. The fragmentation may or may not result in population subdivision, but will likely affect the carrying capacity of the population as the habitat area is decreased [5]. Also, if the species is territorial there may be fewer territories available. In addition, vital rates and behavior of the individuals may also be affected, e.g., due to edge effects [6]. The home range of each individual in the fragmented landscape may include multiple fragments, forcing the individual to move among them through the human-modified landscape. Depending on the characteristics of the non-habitat (i.e., "matrix") areas of the landscape, this may result in higher levels of human-wildlife conflict and higher mortality due to hunting, poaching and collisions with vehicles [7]. From the perspective of species, like jaguar, that have very large home ranges, most habitat fragmentation would be fine-grained [8–12]. The impact of such fragmentation may not be loss of populations, but a more subtle interaction between habitat fragmentation and road mortality such that populations with more fragmented habitat suffer higher mortality.

In this paper, we analyze the factors affecting the viability of a top predator in a fine-grained fragmented landscape. The use of selected species as a basis for site-based conservation has been widely used for designing landscape conservation (e.g. [13–15]), building on the concept of umbrella species, whose protection indirectly causes the protection of several other species. Large carnivores, such as jaguars, can act as umbrella species because of their large area requirements [16]. In addition, as top predators they play an important role in maintaining healthy ecosystems [17].

Remaining jaguar (*Panthera onca*) populations are becoming increasingly fragmented and isolated throughout the species’ range [18]. The Upper Paraná River, in the Brazilian Atlantic Forest region, provides a unique opportunity to study jaguars in a landscape that includes highly fragmented areas as well as relatively intact ones. Jaguar populations may exhibit a metapopulation structure, and an important step in assessing the status of jaguars is to determine the spatial structure of its populations in this region. The Pontal do Paranapanema Region, together with the upper Rio Paraná ecosystem still maintains approximately 50,000 km^2^ of relatively connected and well-preserved semi-deciduous Atlantic forests and marshlands, surrounded by a mosaic of agriculture, extensive cattle ranching, and agrarian settlements [19]. It is among the few areas where large carnivores such as jaguars, pumas and ocelots persist [20]. There are several protected areas in the region, including the Morro do Diabo and Ivinhema state parks in Brazil, Ilha Grande national park and Iguaçu national park in Brazil and Argentina. In this region, jaguar is identified as one of a handful of umbrella species whose long-term viability is a conservation priority [1,21]. In the broader context of the species' range, the populations in the Upper Paraná ecosystem are considered to be of highest conservation concern [22]. These populations are threatened by several factors, including habitat loss, habitat fragmentation, and consequential mortality from interactions with livestock [9,19,23–25] and proximity to roads. These populations are also relatively isolated from populations in other regions such as the Pantanal and the Coastal Atlantic forests [8].

We developed a habitat-based metapopulation model to estimate the viability of jaguars in the Upper Paraná Region and the contribution of road mortality to the risk of decline of this species. We analyzed the simulation results to identify interaction between the effects of road mortality and habitat fragmentation. We interpreted these results in terms of landscape management and corridor restoration in a human-dominated landscape, with the hope that this approach will contribute to future state and national government efforts and well-founded conservation policies in the Upper Paraná ecosystem, and provide the basis for long-term landscape conservation planning within the region.

## Materials and Methods

### Overview of the model

**Fig 1:**
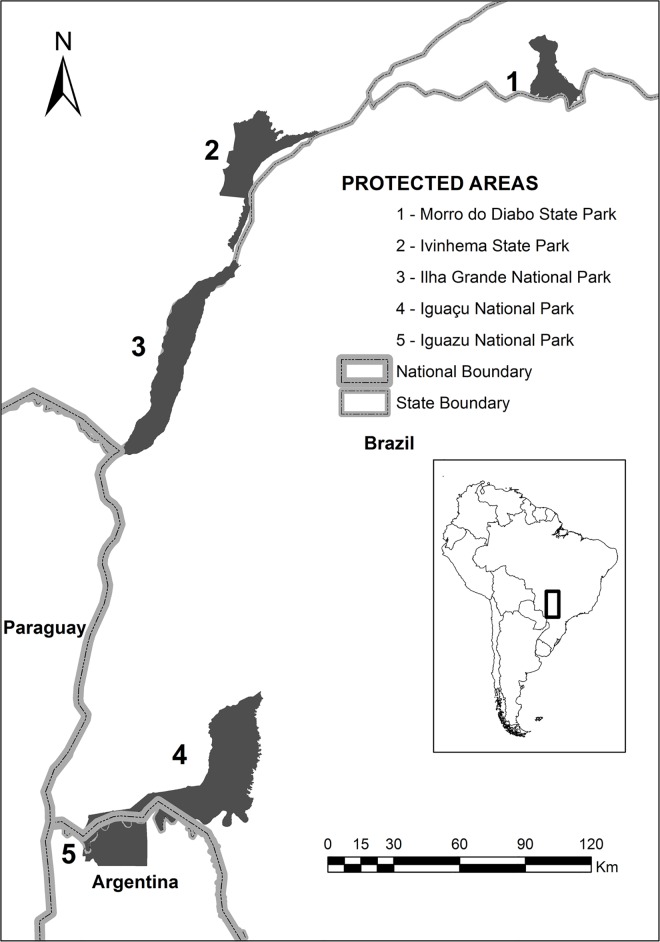
Some important protected sites in the study area, along the upper and the lower Paraná River. The inside frame indicates the location of the study.

We modeled the metapopulation dynamics of the jaguar in an approximately 340 km x 660 km region of the upper Paraná-Paranapanema region, along the Paraná and Paranapanema Rivers in Brazil (Fig 1). We developed the model using the software RAMAS GIS [26], which uses spatial data (such as habitat maps) to determine the spatial structure of a metapopulation (i.e., number and location of its populations) and simulates metapopulation dynamics with an age- or stage-structured (matrix) model for each population. RAMAS GIS can incorporate stochasticity, density dependence, and other factors, and has been used to analyze the viability of a variety of species (e.g., see [27]), including large carnivores such as ocelot [28], cougar [29], Florida panther [30], grizzly bear [31] and Iberian lynx [32].

We developed a habitat model using data on land cover and habitat selection of jaguars, and used this model to calculate the spatial structure of the metapopulation. We used a combination of our own data and data from the literature to estimate demographic parameters and combined these parameters with the spatial structure to build a stage-structured, stochastic, spatially explicit metapopulation model. Finally, we used this model to simulate the dynamics of the jaguar metapopulation and to estimate its viability under various scenarios. The components of the model are detailed below.

### Habitat Model

The spatial structure of the jaguar metapopulation in the upper Paraná-Paranapanema ecoregion was based on a habitat map that we developed using logistic regression. Presences included 1223 locations at which jaguars were recorded using a variety of methods including radio collars, camera traps, animal tracks, and personal observations. Predictor variables included land cover maps with 7 categorical variables, which we converted to percentages by reducing the spatial resolution. The most important variables were primary and secondary forest and marshlands. Information about the data and the maps that formed the basis of this analysis, and the details of the habitat model are described in S1 Appendix.

We used the habitat map to determine the spatial structure of the metapopulation, including size and location of main habitat patches and the distances between them. The link between the habitat map and the jaguar metapopulation was characterized by two parameters. *Neighborhood distance* was used to identify nearby grid cells that belong to the same patch (i.e., population) and may represent the mean foraging distance of the species or the size of the home range. Based on the average home range area of 125 km^2^ [33], the diameter of a circle shaped home range was calculated as 47 cells (1 cell = 270 meters), which was used as the *Neighborhood distance* parameter. *Threshold habitat suitability* is the minimum habitat suitability value below which the habitat is not suitable for reproduction and/or survival. As the threshold is increased, the number of populations identified by the program increases. We adjusted this parameter so as to identify the four populations that were determined to be genetically distinct populations by Haag and colleagues [34] as separate populations of the model. This was achieved by setting *Threshold habitat suitability* to 0.75. The proportion of the study area with habitat suitability (HS) at or above 0.75 is 3.8%. This represents a conservative (precautionary) value, because only a small portion of the landscape is assumed to be suitable.

Based on the habitat map and these two parameters, RAMAS GIS [26] identified the spatial structure of the metapopulation (i.e., the size and location of habitat patches that support populations). Each patch supports one population (or "subpopulation") of the metapopulation. This method of patch identification is described elsewhere ([35], [36]).

### Carrying capacity and Initial Abundance

After the populations (habitat patches) were identified, the carrying capacity (K) and initial number of individuals were calculated for each patch, using the total habitat suitability (HS) value of each patch (see [26,36] for a detailed description). Based on home range sizes and the camera trapping results [33], the carrying capacity was estimated for the Morro do Diabo population as 13 animals (including only adults and sub-adults of both sexes). The carrying capacity was then scaled to the other populations based on the total HS of the Morro do Diabo population (i.e., the sum of the habitat suitability values of all grid cells identified by the model as belonging to this population), which was 3311. Thus, the carrying capacities of the other populations were calculated by multiplying their total HS value by 0.003926 (13/3311). We excluded fragments with a carrying capacity of 2 or less. Initial abundance was assumed to be equal to carrying capacity for all populations, and distributed by age classes according to a stable age distribution (SAD). Carrying capacity in this model is the equilibrium population size under density dependence (see below). Although a recent, major disturbance could have reduced the abundance below the carrying capacity (and pushed the age distribution away from SAD), there is no evidence for such an event.

### Demographic Structure and Vital Rates

We developed a stochastic, age- and sex-structured model with 15 annual age classes for each sex; thus a total of 30 age classes. We based the main demographic parameters of the model on the model developed in a workshop [37] in which experts reviewed all the available field and captive data on jaguars and related species. Although the model is based on data from a large variety of sources, data are not available to estimate all parameters independently. Thus, the model includes a number of simplifying assumptions—equal sex ratio at birth, fecundity independent of age after age 3 (the age of first reproduction), annual survival (*S*) in ages 3 to 10 is the same—which reduce the number of parameters.

The matrix model is parameterized according to pre-reproductive census. Thus, we calculated fecundity (i.e., the elements of the matrix representing reproduction) as *F* = *m*·*S*_0_, where *S*_0_ is survival rate from birth to age 1, and *m* is the number of cubs per female per year. We assumed the same survival rates as used in the workshop report [37], and we set *m* to 1.0, assuming a litter size of 2, and that only 50% of females breed at any one year (because of birth interval of 2 years). We then divided the resulting fecundity into fecundity representing daughters and sons. The population growth rate (i.e., the finite rate of population increase as determined by the eigenvalue of the matrix) is 1.06, representing population growth of 6% per year. We analyzed the sensitivity of results to the matrix by changing survival rate by ±0.03 and fecundities by ±0.05 such that the population growth rate ranged from 1.025 to 1.095 (i.e., 2.5% to 9.5% per year).

### Density Dependence

**Fig 2:**
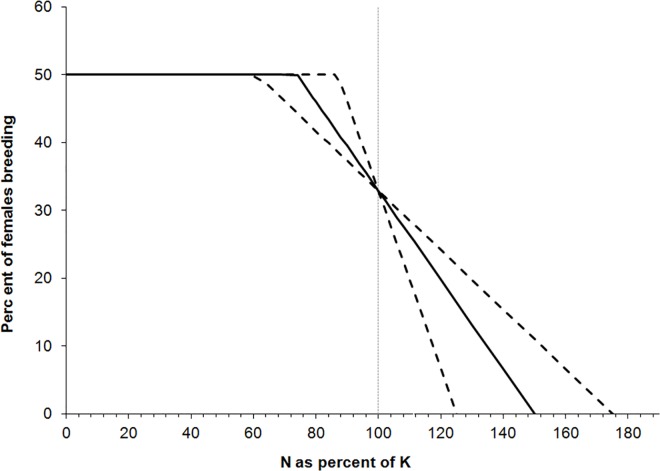
Density dependence function used in this model (solid line) and its uncertainty limits used in the sensitivity analysis (dashed lines). In all models, the proportion of females that are breeding is 50% when population size (N) is small relative to carrying capacity. At carrying capacity, the proportion breeding is 32.9% (which gives an eigenvalue of 1.0; see text for details). The percent breeding declines as N increases, dropping to zero when N = 1.5∙K (1.25 to 1.75 used in the sensitivity analysis).

We assumed that at high densities, the proportion of females breeding decreases as a function of the ratio of population size to carrying capacity [37]. However, we used a function that is different than the one used by [37], who assumed that the proportion changed according to the function (50-((50–40*((N/K)^15))). We changed this function for two reasons. First, it results in 40% of females breeding at carrying capacity. This percentage results in a population growth rate of 1.027. However, by definition, growth rate at carrying capacity should be 1.0 (i.e., no population growth or decline), which in this case can be obtained by setting the percentage of females breeding to 32.9% at carrying capacity. Second, the function used by [37] represents an extreme form of density dependence, in which the proportion of females breeding decreases to 0 when the population size is only about 11% over the carrying capacity (Fig 2). Instead, we used a function in which this proportion declined linearly for N/K>0.8, such that it reached 0 only when N/K>1.5 (Fig 2). In other words, breeding does not cease until the population size increases 50% beyond the carrying capacity. We analyzed the sensitivity of results to this function, by varying it between the uncertainty limits depicted in Fig 2.

### Dispersal Rates

Dispersal rate between populations was modeled as a negative exponential function of distance, with distances between populations measured from the center of the source population to the edge of the target population. Centre-to-edge distances are used to model asymmetric rates of dispersal expected between two habitat patches that are substantially different in size [38]. The function was parameterized based on information from [39] which calculated dispersal rates among some of these jaguar populations. From 3 populations in close proximity to each other, the total dispersal (emigration) rate from each population averaged about 20% per generation, corresponding to about 2.5% per year. Thus, we set the dispersal-distance function such that the total dispersal from each population (i.e., sum of all dispersal rates from this population to all other populations) ranged from 1.9% to 3.9%, with an overall average of about 2.5%. This resulted in a dispersal matrix with 0.0 to 1.5% dispersal rate between pairs of populations (Table A in S2 Appendix). Dispersal was modeled as a density-dependent process for each population, with the dispersal rate being directly proportional to population size. Under density-dependent dispersal, when the population size (*N*) is lower than the carrying capacity (K), the proportion dispersing is lower in proportion to the ratio of *N*/*K* [40]. We analyzed the sensitivity of results to dispersal rates by changing them by ± 100% (i.e., from no dispersal to twice the values in Table A in S2 Appendix). Because of lack of quantitative information about age- and sex-specific dispersal rates, we assumed an equal probability of dispersal from all stages.

### Stochasticity

Environmental stochasticity was modeled by sampling mortality (1-*S*), fecundity and dispersal rates from log-normal random distributions with coefficients of variation of 20% (we sample mortality instead of *S* to eliminate truncation of sampled values over 1, because most S values in this model are close to 1; see [26]). This value of coefficients of variation is similar to the variability of mortality included in the models of [37] and [41]. We analyzed the sensitivity of results to variability by changing the standard deviations of vital rates by ±20%.

An important source of environmental fluctuations is frequent fires, which are more common in years with hotter and drier weather patterns. Because such patterns have the potential to affect many populations simultaneously, environmental fluctuations are expected to be spatially correlated to some extent (such that different populations experience partially synchronous temporal fluctuations). However, the correlation would not be perfect, because there are other, more local, sources of environmental variability, such as hunting and other human disturbances. Thus, we assumed environmental fluctuations to be moderately correlated among populations, based on a correlation-distance function (see [26,36]) that resulted in correlation coefficients ranging from 0.09 (for pairs of distant populations) to 0.76 (for pairs of nearby populations). In addition, demographic stochasticity was used by sampling the annual number of survivors and dispersers from binomial distributions and the number of offspring from Poisson distributions [42].

### Road Mortality

**Fig 3:**
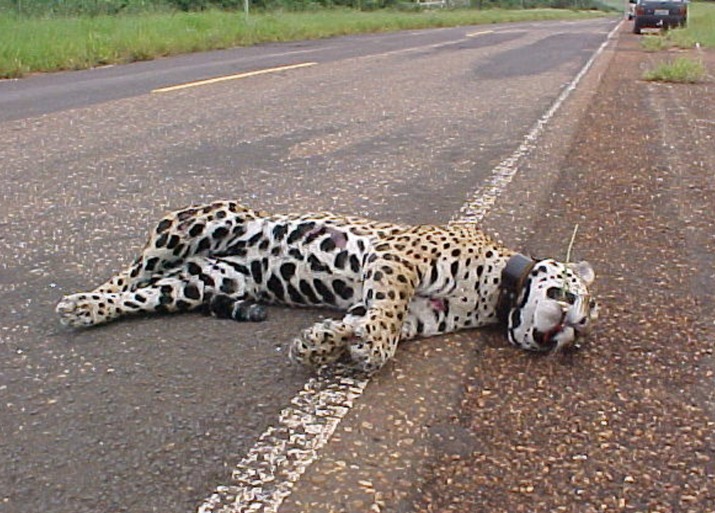
Radio-collared female jaguar (*Panthera onca)* roadkill, Morro do Diabo State Park, Brazil.

There is very little information about mortality, except for anecdotal reports of jaguars killed in various populations, and an estimate of about 1 jaguar killed per year by vehicles in the Morro do Diabo population (based on anecdotal reports and observations of road kills by LC and FL during daily routine around the only road that crosses the park) (Fig 3). In addition to vehicle strikes, roads also allow easier access for hunters to parts of the species' range. This can result in direct mortality of jaguars by poaching or indirect mortality because of the competition with hunters for prey species. We assumed that population-specific road mortality from all these causes (percent of a population killed because of roads) is proportional to the product of the number of linear km of road per jaguar, and human density (which we used as an index of traffic) within the minimum convex polygon around all the habitat patches (fragments) that form that population. Based on this assumption and the estimate mentioned above, we calculated the expected road mortality for all populations. Data on human population density were from the Global Rural-Urban Mapping Project (version 1, [43]). Road lengths were calculated from the Vector Map Level 0 (VMAP0) road layer available through the United States National Imagery and Mapping Agency. We analyzed the sensitivity of results to road mortality by changing the percent mortality by ±100% (i.e., from no mortality to twice the values in Table 1). We assume that the estimates of annual survival used in the model (see above) incorporate sources of mortality other than road mortality, such as natural mortality and poaching.

**Table 1:** Properties of jaguar populations in the upper Paraná-Paranapanema, Brazil. Populations were identified by RAMAS GIS (see Fig 4).

	Area km^2^						Road Mortality	
Population	Core (habitat)	Minimum convex polygon (MCP)	Total road length in MCP (km)	Human population density in MCP (km^-1^)	Carrying capacity (K; number of jaguars)	km road per jaguar at N = K	Percent killed	Number killed at N = K
1. Três Lagoas	190	1,615	81	11.8	8	10.1	8.5%	0.7
2. Rio Pardo	145	8,029	741	1.6	6	123.5	13.9%	0.8
3. Ivinhema-Ilha Grande	1,121	16,536	1,143	13.0	48	23.8	22.2%	10.7
4. Morro do Diabo	292	674	90	15.5	13	6.9	7.7%	1.0
5. Itabo-Carapa	201	3,591	87	10.0	9	9.6	7.0%	0.6
6. Morombi	507	11,833	555	18.0	22	25.2	32.7%	7.2
7. Green corridor	5,1	17,288	922	24.6	231	4.0	7.0%	16.3
8. San Rafael	796	2,952	104	17.0	34	3.1	3.8%	1.3
Overall	8,353	62,518	3,722		371	10.0	10.4%	38.5

### Simulations, Scenarios, and Sensitivity Analysis

We used a series of simulations to analyze the dynamics of the jaguar population using the habitat-based metapopulation model described above. Each simulation consisted of 1,000 replications and each replication projected the abundance of each population for 100 years, which corresponds to 13–14 generations. To assess the effects of fragmentation through road mortality, we ran simulations with several different levels of mortality, including 0%, 100% and 200% of the mortality calculated as described above. We analyzed simulation results in terms of final metapopulation size (total number of individuals in all populations in year 100, averaged over the 1000 replications), population persistence (number of years a population was extant, i.e., included at least 1 individual, averaged over the 1000 replications), and expected minimum metapopulation size (the minimum metapopulation size during the 100-year projection interval, averaged over the 1000 replications).

We analyzed the sensitivity of these results to parameter uncertainty, focusing on the five sets of parameters mentioned above: the stage matrix (survival rates and fecundities), the density dependence function, dispersal rates, environmental variability, and road mortality. We sampled the values of these 5 sets of parameters from uniform random distributions, creating 1000 models. These distributions represent the uncertainty of the parameters due to lack of information or measurement error, not their natural variability (natural variability is discussed above, under *Stochasticity*). Each of these 1000 models were run as described in the previous paragraph (with 1000 replicates each). We summarized the uncertainty as the interquartile range (IQR; the range from the 25th to 75th percentiles) of the results of these 1000 models.

## Results

### Patch Structure

**Fig 4:**
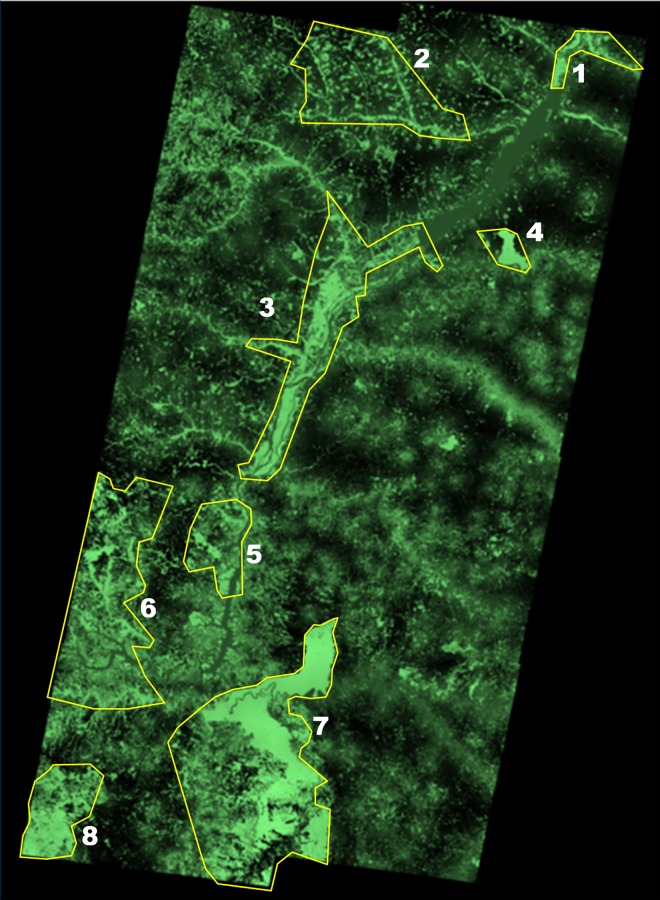
Spatial structure of jaguar populations identified by the model in the upper Paraná region. Lighter shading indicates greater habitat suitability as given in Figure A in S1 Appendix. The polygons outline the populations. The population numbers correspond to those in Table 1, and in Figs 5 and 6.

The habitat model produced the habitat map (see Figure A in S1 Appendix), which was validated using ROC curve (see Figure B in S1 Appendix). We tested a sample of occurrence locations or presences (that were not used for model building) with the map projected to the most recent classification of land cover. This resulted in an omission error rate (false negative rate) of 0.01 at a habitat suitability threshold of 0.75 (3 out of 302 occurrence records). Based on the habitat map resulting from this model, RAMAS GIS identified the spatial structure of the metapopulation as 8 populations (i.e., 8 clusters of suitable cells within the neighborhood distance of each other) with a total habitat area of 8,353 km^2^ and a total carrying capacity of 371 individuals (Fig 4, Table 1). The patch structure was realistic considering the remaining habitat, known jaguar occurrences and the location of some protected areas in the upper Paraná-Paranapanema region.

### Road Mortality

Based on the estimate of 1 jaguar killed in Morro do Diabo per year, and the assumption that road mortality is a linear function of the road length per jaguar and human density, we estimated overall annual road mortality as 10.4% of the population, resulting in about 39 jaguars killed per year at carrying capacity (Table 1). Estimated annual road mortality varied from 4% to 33% for different populations, and was largest for populations with higher number of km of road per jaguar (esp. Rio Pardo, Ivinhema and Morombi; Table 1).

### Population Size and Trends

At the end of the 100-year simulation, the average predicted metapopulation size was about 197 individuals (IQR 105–299), down from an initial size of 371, representing a decline of about 47% (19% - 72%). The average number of extant populations (occupied patches) was 5.7 (4.5–7.1), down from 8. Expected minimum metapopulation size was about 155 individuals (82–236). Additional results of the sensitivity analysis are presented in S2 Appendix.

### Effect of Road Mortality on Population Dynamics

**Fig 5:**
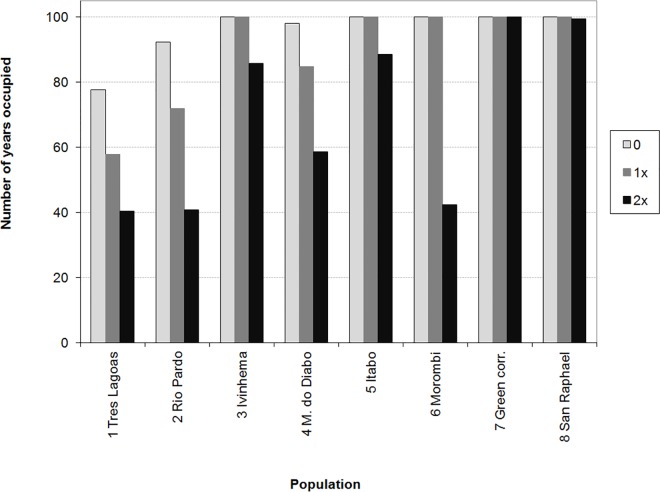
Impact of road mortality on jaguar population persistence: Number of years out of 100 that each population was extant, under no road mortality (0; light gray bars); estimated mortality (1x; dark gray bars); and twice the estimated mortality (2x; black bars).

**Fig 6:**
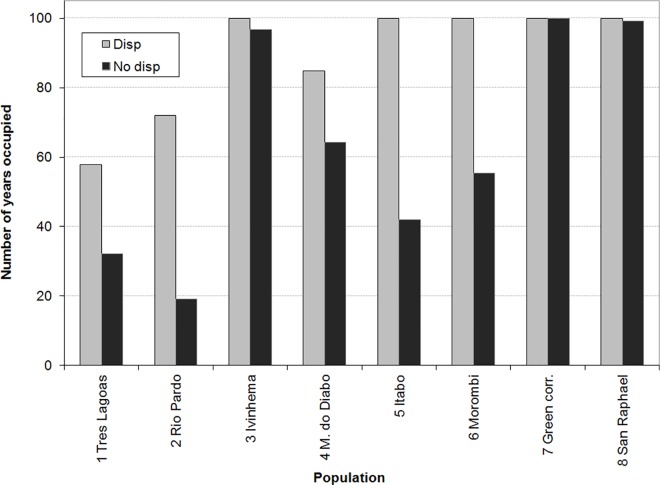
Effect of dispersal on population persistence: Number of years out of 100 that each population was extant under estimated mortality. For several populations, model with no dispersal (black bars) resulted in much lower persistence than model with dispersal (gray bars).

Road mortality affected the population size and persistence (Table 2; Fig 5). Changing road mortality rate from 0 to the rates in Table 1 ("1x" in Table 2 and Fig 5) and to twice these rates ("2x") resulted in about 80% reduction in final metapopulation size and expected minimum abundance, and 45% reduction in the number of extant populations in year 100 (Table 2). Population persistence was substantially reduced for populations 1 (Tres Lagoas) 2 (Rio Pardo) and 6 (Morombi) due to road mortality (Fig 5). For these populations, persistence was strongly sensitive to dispersal: a model with no dispersal resulted in much lower persistence than the model with dispersal (Fig 6).

**Table 2:** Effects of road mortality rate for jaguar population.

	Road mortality rate*		
	0	1x	2x
Metapopulation size at year 100	351	226	73
Number of extant populations at year 100	7.5	6.6	4.1
Expected minimum abundance	321	173	55
Total road mortality** (average)	0	1062	642.3
Total road mortality (range)	0–0	514–1608	376–980

* Road mortality rate is the percentage of jaguars killed; 0: no road mortality; 1X: the percentages in Table 1; 2X: twice the percentages in Table 1.

** Total road mortality is the number of jaguars killed over 100 years in all populations, averaged over 1000 replications of the model with mid values of all parameters other than road mortality rate.

## Discussion

Our results indicate that jaguars in the upper Paraná-Paranapanema region exist in eight populations with varying sizes and subject to varying degrees of human disturbance. Road mortality is likely impacting the jaguar populations in this region, decreasing both the abundance and the distribution of the species. Our analysis assumed that populations most affected by road mortality would be those with more roads and higher human populations. However, in our model, these factors interacted with the fragmented habitat such that populations in more fragmented areas are more impacted by road mortality. This is because in these populations (e.g., population 2, Rio Pardo; and population 6 Morombi), the fragmentation of habitat results in lower density of habitat (see Fig 4) and consequently lower density of jaguars. Thus, a population in highly fragmented habitat would have higher density of road per jaguar (compared to populations in less fragmented habitat), even if the road density per unit area is the same as in other populations. For example, in population 6, Morombi, road density is about 0.047 km^-1^, less than the overall average of 0.06km^-1^ for all populations. However, because of fragmented habitat, jaguar density is low, and km road per jaguar is 2.5 times the overall average (Table 1). The nature of this interaction between habitat fragmentation and road mortality is determined by the way jaguars perceive the pattern of fragmentation. Because of the large home range size of jaguars, the fragmentation (especially in population 2 and parts of population 6 and 7) is fine-grained from the perspective of this species. These findings corroborate one of the main conclusions of a comprehensive review on road impacts on animal populations[44] that species "with large movement ranges, low reproductive rates, and low natural densities" (a profile that jaguar fits well) would be negatively impacted by roads, regardless of their behavioral response to roads.

Previous research has shown that impact of roads may be at least as great as the impact of habitat loss [45]. Our study shows that habitat loss (which often results in fragmentation of remaining habitat) and road mortality may interact synergistically such that road impacts are higher in fragmented landscapes. In extreme cases, the interaction between road mortality and fine-grained habitat fragmentation may lead to source-sink dynamics, whereby populations with highly fragmented habitat are maintained only by dispersal from populations with less fragmented habitat. There is some indication in our model results that this may already be happening in the upper Paraná-Paranapanema region. The populations with the most fragmented habitat remained extant for much less of the simulated 100 years when the model included no dispersal (Fig 6), indicating that the persistence of these populations are dependent to a large extent on dispersal from other populations. The possibility of source-sink dynamics must be considered when evaluating conservation options for species in fragmented landscapes. For example, even though increasing connectivity by developing and maintaining corridors in general can increase the viability of species, they can also exacerbate the negative effects of source-sink dynamics on species viability.

Our study demonstrates the utility of linking habitat and demographic models to assess impacts on species living in fragmented landscapes, as well the importance of assessing impacts with models at different spatial scales. Although range-wide models based on habitat (e.g., [8]) are useful in planning overall conservation strategies, effects and interactions at the local level (such as the interaction between habitat fragmentation and road mortality) are better analyzed with models that combine habitat and demography.

The threats, ecology, distribution, and management options of the jaguars in the Upper Paraná Paranapanema region necessitate the use of models that combine habitat and demography. In this study, we focused only on road mortality and its interaction with habitat fragmentation. However, jaguars in this region are threatened by several factors that we did not explicitly model, such as habitat loss and mortality resulting from their interaction with livestock. Each of these factors affects a different aspect of the jaguar metapopulation. Incorporating these factors into our model would require developing scenarios of future land use, based on past trends and patterns of land-use change, current ownership maps, and plans for future development. Although such information is not available at the moment, when it becomes available, models that link habitat and demography, such as the one presented here, will allow assessing the cumulative effects of these threat factors. Similarly, a number of assumptions in our model (such as equal dispersal rates for all stages) were necessitated by lack of quantitative information. When relevant information becomes available, it can be used to modify these assumptions. However, we believe that such improvements to the model would not alter our main conclusions about the interacting effects of road mortality and fine-grained fragmentation.

There are several types of management actions that may benefit these populations, including habitat protection, increasing connectivity, decreasing road mortality, habitat enhancement or restoration [8–12,19,41], which can also be studied with the type of spatially explicit model we developed. For example, an evaluation of habitat management options can be based on the eight large suitable patches identified in this study, which together were about 8,400 km^2^ in area or equivalent to 4% of the potential habitat in the study area. Such an evaluation would require overlaying the habitat map we developed in this study with detailed maps of land ownership (and availability for conservation). This would allow identifying options for establishment of new protected-areas at different intensities of management (such as intensive use areas, buffer zones or intermediate use areas, strictly protected areas, and wildlife corridors). Each option would then be simulated as a set of changes to the spatial structure of the model, and each option would be evaluated in terms of the viability of the species. The viability result for each option can also be combined with the cost of that option, if such cost information is available (see [46] for an example of such an analysis). We believe that the results of such a detailed analysis could lead to specific recommendations for habitat conservation actions in this human-dominated landscape.

## Supporting Information

S1 Appendix Habitat Suitability Model for Jaguar in the Upper Paraná River Corridor. (PDF)

S2 Appendix Sensitivity Analysis. (PDF)
