# Supplementary material for: Implications of Fine-Grained Habitat Fragmentation and Road Mortality for Jaguar Conservation in the Atlantic Forest, Brazil
Source: PLoS One. 2016 Dec 14;11(12):e0167372. doi: 10.1371/journal.pone.0167372 (PMC5156341; doi:10.1371/journal.pone.0167372)
Supplement: S1 Appendix — (PDF) [file pone.0167372.s001.pdf]

"Implications of Fine-grained Habitat Fragmentation and Road Mortality for Jaguar Conservation in the Atlantic Forest, Brazil" by Laury Cullen Jr., Jessica C. Stanton, Fernando Lima, Alexandre Uezu, Miriam L. L. Perilli and H. Reşit Akçakaya.

S1 Appendix: Habitat Suitability Model for Jaguar in the Upper Paraná River Corridor

The habitat map provides the basis for the spatial structure of the jaguar metapopulation in this region. We modeled habitat suitability based on the land cover where Jaguar occurrences were recorded in the landscape. Occurrence locations used to build the suitability model were recorded from a combination of radio collar data, camera traps, animal tracks, and personal observations.

Land cover classification was done by one of the authors (A.U.), using supervised classification. Two land cover maps were available both with 30m resolution, but with different spatial extents, representing the land cover in approximately the years 2003 and 2008. These maps are mosaics of six and eight rectified Landsat scenes, respectively. The 2003 land cover map extends 50 km to each side of the Paraná and Paranapanema Rivers from approximately 22˚31' S; 52 10'W in the North to approximately 25˚21'S; 54˚28'W in Brazil. This landscape coverage will be referred to as the 'past land cover'. The 2008 map covers a larger spatial extent including portions of Paraguay and Argentina. This map will be referred to as the 'present land cover' and forms the basis for the spatial extent of this study.

The majority of the jaguar occurrence observations were made prior to 2007. In order to map the habitat in the present land cover configuration without sacrificing the richer occurrence data available prior to 2007, we used pre-2007 occurrence locations with the past landscape map to build the model, then projected the model to the broader extent of the present land cover. 1223 occurrences locations were used for model building.

The land cover classification categories were slightly different between the two coverages. In order to build the model in one timeframe and project it to another it was necessary to reconcile the differences in land cover classifications between the two maps. The land cover categories of the past and present land cover maps are presented in Tables A and B. To reconcile the land cover categories, we made as few changes as possible while trying to avoid introducing error by making assumptions about the landscape. In the past land cover map, Dense Marsh and Open Marsh were combined into a single Marsh classification. In the present land cover map the following changes were made: Mature Forest was assumed to represent a roughly equivalent land cover class as Primary Forest in the past land cover map

and so it was simply renamed; Agriculture and Sugar Cane were combined into a single Agriculture classification; and Exposed Soil, Reforested, and Urban were left out of the analysis since there were no equivalent land cover categories in the past land cover map.

Table A. Proportion of land cover on the study area in the past (prior 2007).

Classification	Percent of total	Reclassification used in the analysis
Water	7.0	Water
Primary Forest	5.2	Primary Forest
Secondary Forest	2.6	Secondary Forest
Alluvial Forest	1.0	Alluvial Forest
Dense Marsh	5.0	Marsh
Open Marsh	11.0	
Agriculture	17.2	Agriculture
Pasture	51.0	Pasture

Table B. Present proportion of land cover on the study area.

Classification	Percent of total[a]	Reclassification used in the analysis
Water	7.2	Water
Mature Forest	5.7	Primary Forest
Secondary Forest	6.4	Secondary Forest
Marshlands	8.7	Marsh
Alluvial Forest	0.2	Alluvial Forest
Reforestation (Eucaliptus+Pinus)	0.2	*not used*
Agriculture (soya + corn + wheat)	9.1	Agriculture
Sugar Cane	8.3	
Pasture	36.3	Pasture
Exposed soil/land	17.5	*not used*
Urban	0.4	*not used*

[a] Only area in present land cover map which overlapped in extent with past land cover map analyzed

All of the land cover classes included in the analysis, except for water, were converted to percent variables for use in the habitat suitability analysis by calculating the percentage of each land cover type in a 270 meter by 270 meter grid surrounding each location (this was done by using a 9 pixel by 9 pixel moving window average and then decreasing the image resolution through pixel thinning in a nine pixel by nine pixel window). At this resolution one grid cell is 7.9 ha, still very small relative to the home range area of a single individual. Thus, a 270 meter grid cell is both large enough to convert categorical variables to percentages and small enough to represent jaguar's habitat with sufficient resolution. This technique allows the model to incorporate the landscape immediately surrounding the occurrence record. The Water land cover class was converted to a distance variable by calculating the distance in kilometers from each location to the nearest grid cell classified as water.

Background localities, or pseudo-absences, were chosen randomly from the area that corresponds to the coverage of the past land cover map. A 300 meter buffer was created around each occurrence location and no background points were selected that fell within one of those 300m buffers. There were 4955 background records to choose from.

Logistic regression was used to build a model of landscape suitability. The values of the past land cover variables were paired with occurrences prior to the year 2007 which fell within the coverage of the map. Equal numbers of occurrence and background locations were used for model building. This strategy in model building has been found to result in the convergence of several approaches to model evaluation and threshold selection [1].

Model building was done using a stepwise logistic regression using the MASS package in R [2,3]. This process selects variables for inclusion in the model if they improve the Akaike information criterion (AIC) value. The model starts with all input variables and checks for model improvement by both dropping single variables and adding two-way interactions between variables. The model building process was run several times with different random permutations of the dataset split into training and testing sets (70% training, 30% testing). The intercept and variable coefficients from the model were used to project suitability values to the present land cover. The resulting suitability map was then smoothed using a 7 pixel by 7 pixel moving window average to more realistically reflect the spatial scale at which population processes take place. This resulting habitat suitability map is the

basis for the spatial structure used in the demographic model, which is at the population (not individual) scale.

The coefficients and variables found by the stepwise logistic regression are presented in Table C. The resulting model projected to the present land cover is shown in Fig A. ROC curve and AUC values calculated from 302 additional occurrence locations not used for model building are presented in Fig B for the habitat suitability model projected to the present land cover extent. These results indicate that the developed habitat model provides a robust description of the suitability of habitat for jaguars in this region.

Table C. Variable coefficients in habitat suitability model.

	Estimate	Std. Error	z value[a]	
**(Intercept)**	-0.81	0.24	-3.41	***
**Primary Forest**	2.09	0.43	4.89	***
**Secondary Forest**	2.99	0.55	5.46	***
**Marsh**	1.96	0.32	6.13	***
**Alluvial Forest**	1.41	0.99	1.42	
**Agriculture**	1.46	0.33	4.42	***
**Pasture**	-0.06	0.30	-0.21	
**Water**	-0.22	0.04	-5.47	***
**Marsh * Water**	0.32	0.06	5.23	***
**Primary Forest * Water**	0.26	0.06	3.96	***
**Marsh * Pasture**	-2.20	0.89	-2.48	*
**Alluvial Forest * Agriculture**	16.85	7.27	2.32	*
**Pasture * Water**	0.13	0.05	2.59	**
**Primary * Alluvial Forest**	-12.14	5.25	-2.31	*
**Secondary Forest * Agriculture**	7.40	3.95	1.87	
**Alluvial Forest * Pasture**	-17.40	12.86	-1.35	
**Agriculture * Pasture**	1.33	0.93	1.43	

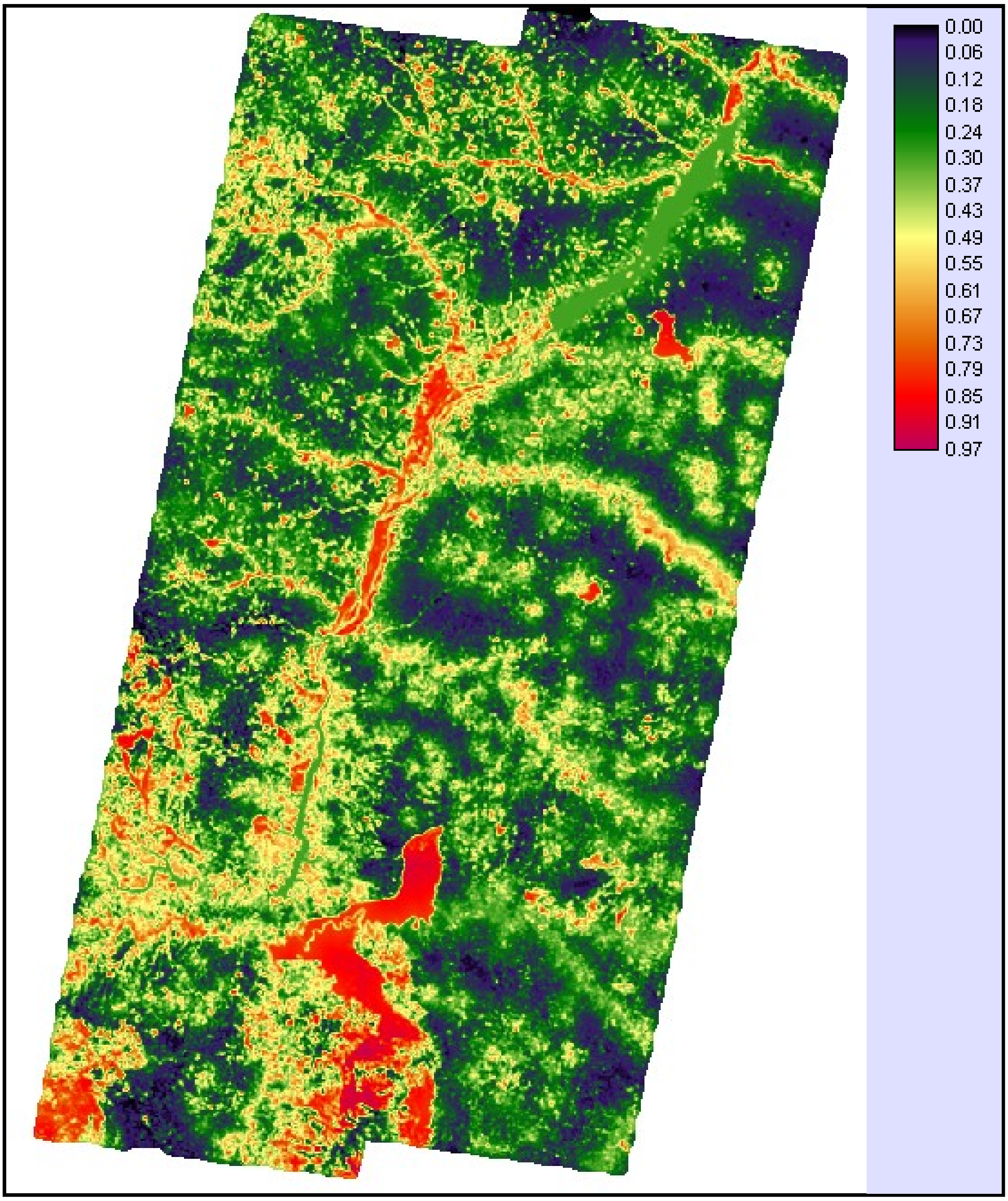

Fig A. Map of Habitat Suitability. Higher values shown in warmer tones represent areas of higher suitability for Jaguar. Lower values shown in cooler tones represent areas of low suitability.

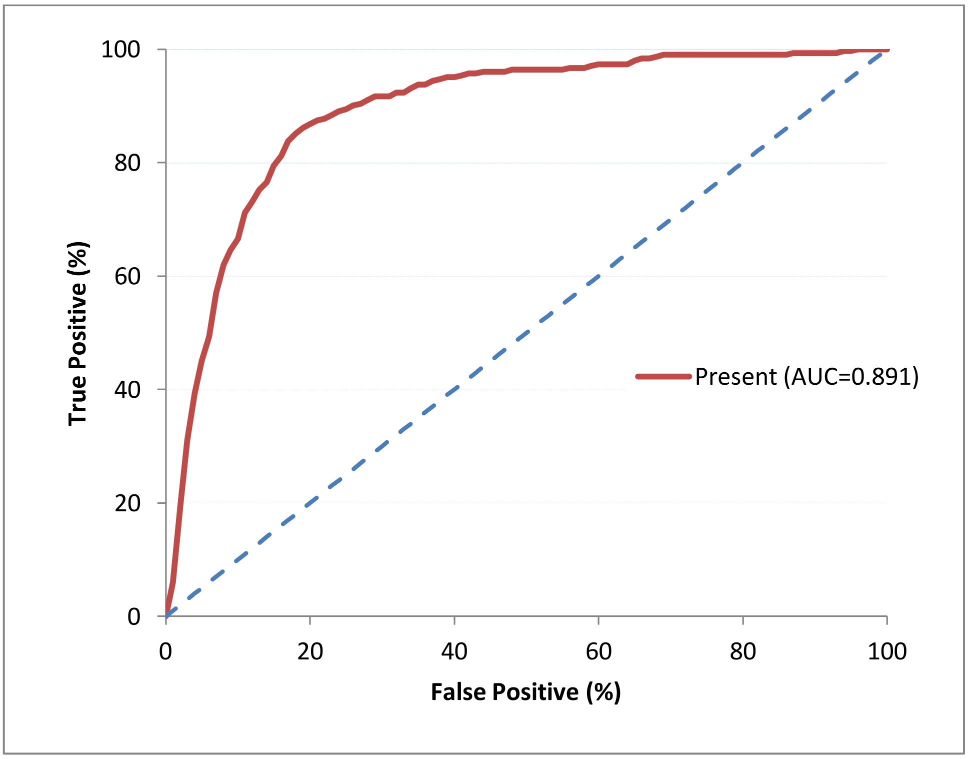

Fig B. ROC curve for the habitat suitability model tested against 302 occurrence locations not used for model building. The red line is the results of the habitat model projected to the present land cover. The blue dotted line shows a theoretical model that performs no better than random.

References

1. Liu C, Berry TP, Pearson RG. Selecting thresholds of occurrence in the prediction of species distributions. Ecography. 2005;28: 385–393.
2. Venables WN, Ripley BD. Modern Applied Statistics with S. Issues of Accuracy and Scale. 2002; 868. doi:10.1198/tech.2003.s33
3. R Development Team. R: A language and environment for statistical computing. Viena, Austria: R Foundation for Statistical Computing.; 2007.
