# Supplementary material for: Implications of Fine-Grained Habitat Fragmentation and Road Mortality for Jaguar Conservation in the Atlantic Forest, Brazil
Source: PLoS One. 2016 Dec 14;11(12):e0167372. doi: 10.1371/journal.pone.0167372 (PMC5156341; doi:10.1371/journal.pone.0167372)
Supplement: S2 Appendix — (PDF) [file pone.0167372.s002.pdf]

"Implications of Fine-grained Habitat Fragmentation and Road Mortality for Jaguar Conservation in the Atlantic Forest, Brazil" by Laury Cullen Jr., Jessica C. Stanton, Fernando Lima, Alexandre Uezu, Miriam L. L. Perilli and H. Reşit Akçakaya.

S2 Appendix: Sensitivity Analysis

We analyzed the sensitivity of model results to uncertainty in five sets of parameters: the stage matrix (survival rates and fecundities), the density dependence function, dispersal rates, environmental variability, and road mortality. We varied the stage matrix parameters such that the eigenvalue ranged from 1.025 to 1.095; we varied the density dependence function between the two functions given in Fig B; we varied dispersal rates from zero to twice the middle values given in Table A; we varied standard deviations of vital rates by ± 20%, and we varied road mortality from zero to twice the values estimated in Table 1. We sampled the values of these 5 sets of parameters from uniform random distributions, creating 1000 models. We used uniform distributions because these distributions represent the uncertainty of the parameters due to lack of information or measurement error, not their natural variability (natural variability is incorporated as stochastic model parameters). We ran each of the 1000 models with 1000 replications, and each replication for 100 time steps (years).

Fig A shows the effect of road mortality on expected minimum abundance (EMA) and population reduction (overall reduction in total number of individuals over the 100-year simulation period). In this figure, each point is one random model, and the results (EMA and reduction) for each point are the average for the 1000 replicates of that model.

Fig B shows the effect of road mortality and dispersal on the number of occupied patches (extant populations) at the end of the 100-year simulation period. Fig C shows the effect of road mortality and stage matrix on population reduction. In both figures, the surface is the fitted second-degree polynomial function. In all figures, "road mortality" is the multiple of the "percent killed" values in Table 1, ranging from 0 (no mortality) to twice the values in Table 1.

Table A. Dispersal matrix used in the model, showing the proportion of individuals moving from each population (columns) to other populations (rows).

	Tres Lagoas	Rio Pardo	Ivinhema - Ilha Grande	Morro do Diabo	Itabo - Carapa	Morombi	Green corridor	San Raphael
**Tres Lagoas**	--	0.6%	0.1%	0.6%	0.0%	0.0%	0.0%	0.0%
**Rio Pardo**	0.9%	--	0.4%	0.8%	0.1%	0.0%	0.0%	0.0%
**Ivinhema - Ilha Grande**	0.4%	0.9%	--	1.3%	1.2%	0.6%	0.3%	0.1%
**Morro do Diabo**	0.5%	0.6%	0.4%	--	0.1%	0.0%	0.0%	0.0%
**Itabo-Carapa**	0.0%	0.1%	0.6%	0.1%	--	1.5%	0.6%	0.3%
**Morombi**	0.0%	0.1%	0.5%	0.1%	1.3%	--	0.8%	0.8%
**Green corridor**	0.0%	0.0%	0.3%	0.1%	0.9%	0.6%	--	0.8%
**San Rafael**	0.0%	0.0%	0.1%	0.0%	0.3%	0.5%	0.5%	--

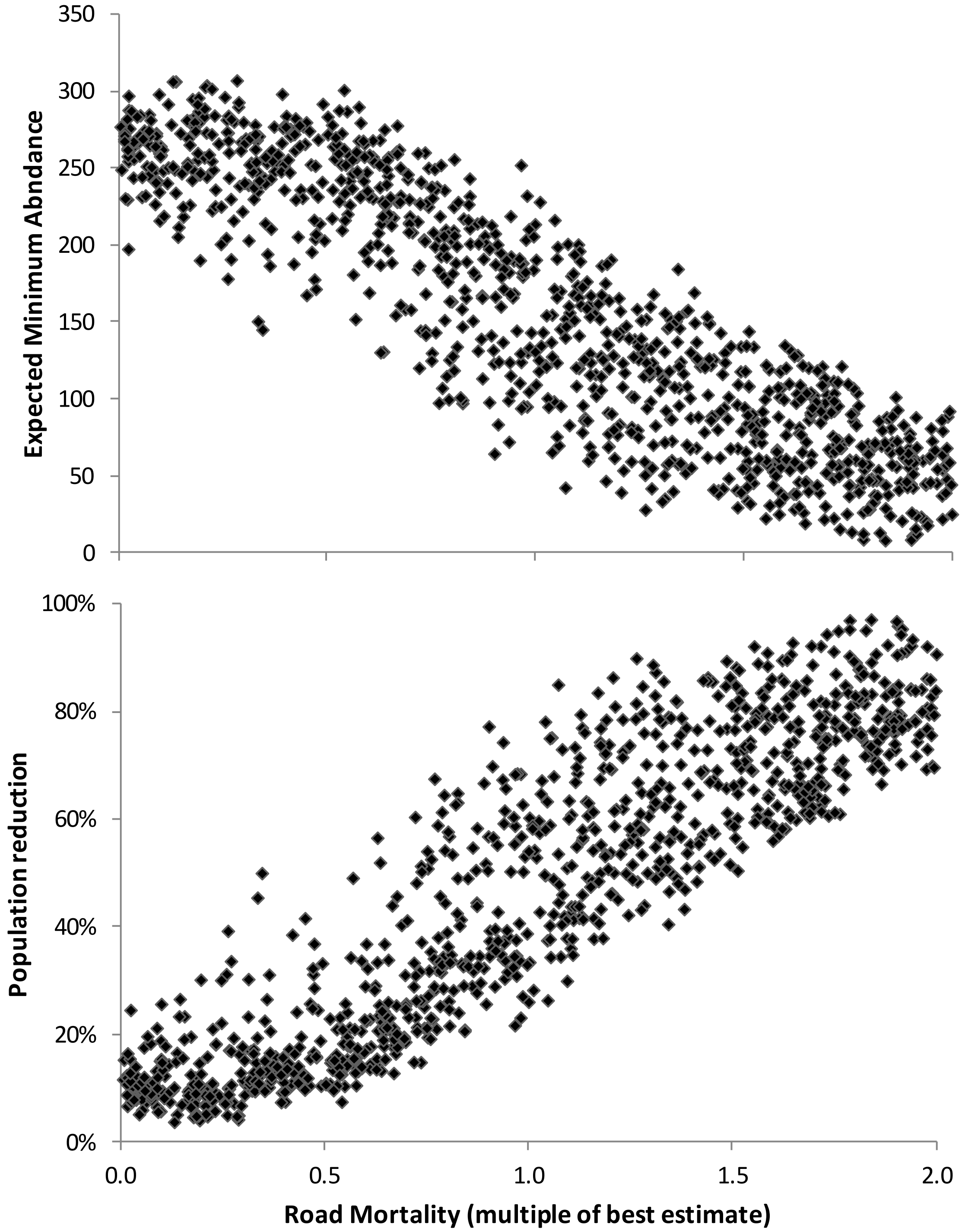

Fig A. The effect of road mortality on expected minimum abundance (EMA) and population reduction (overall reduction in total number of individuals over the 100-year simulation period). Each point represents the results of one random model, and shows the average value of the simulation result for the 1000 replicates of that model. Road mortality is the multiple of the "percent killed" values in Table 1.

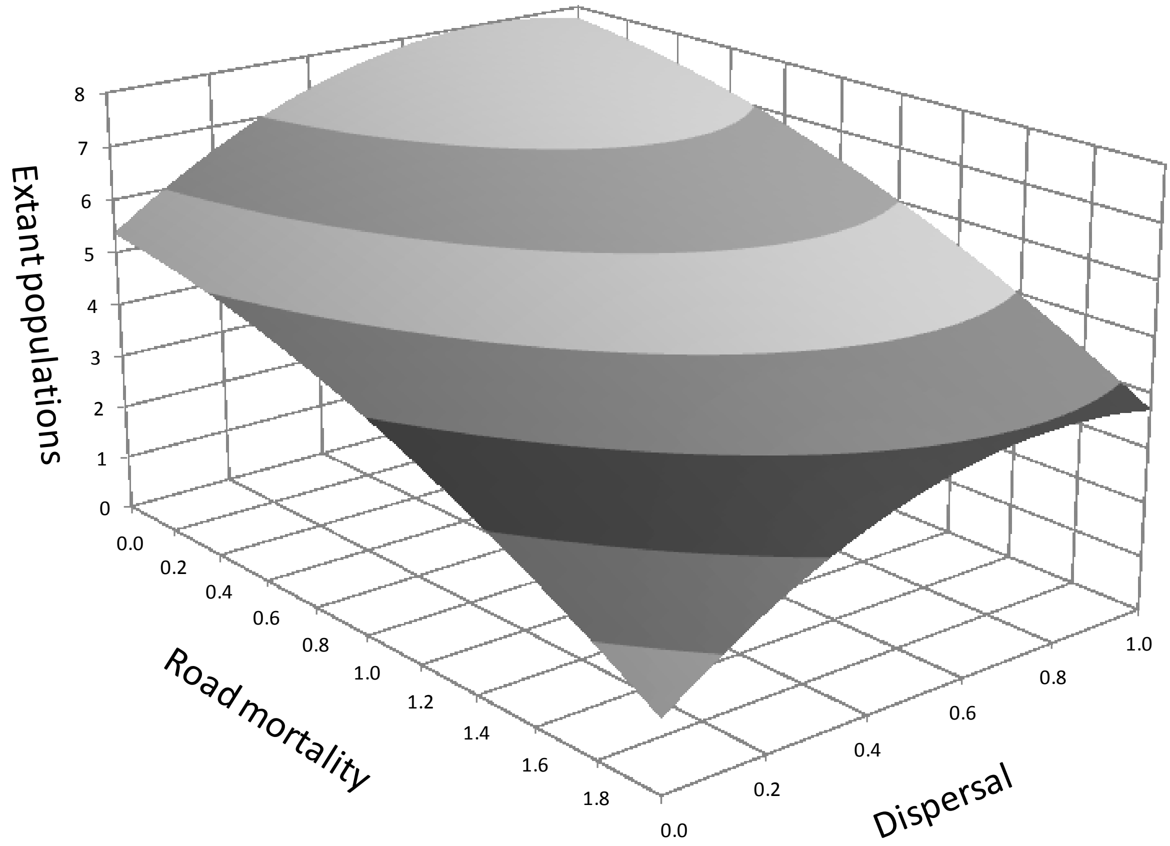

Fig B. The effect of road mortality and dispersal on the number of extant populations) at the end of the 100-year simulation period. The surface is the fitted second-degree polynomial function. The gray zones on the surface indicate levels of the Y-variable, from 1 to 2 populations at the close corner, up to 7-8 populations in the far corner. Road mortality is the multiple of the best estimate (the "percent killed" values in Table 1). Dispersal is the multiple of the values in Table A.

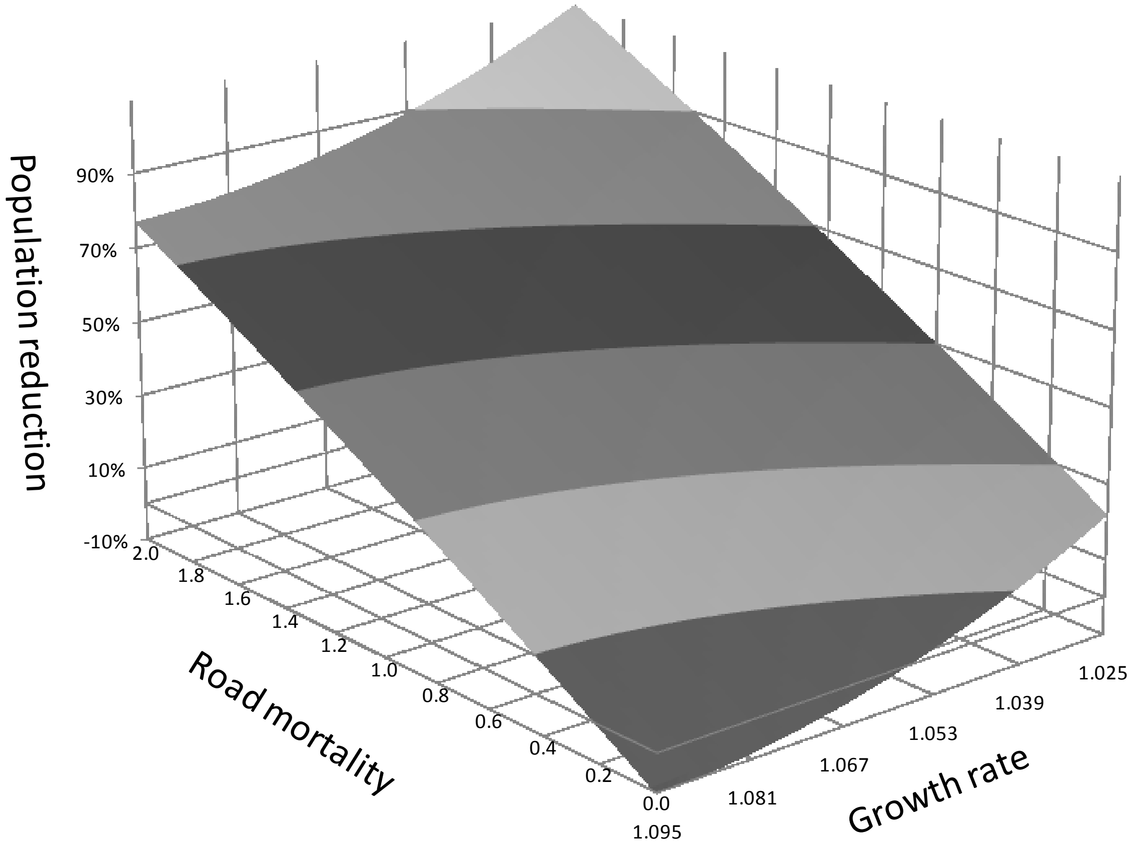

Fig C. The effect of road mortality and population growth rate (eigenvalue of the stage matrix) on population reduction (percent decline in total number of jaguars in all populations during the 100-year simulation period). The surface is the fitted second-degree polynomial function. The gray zones on the surface indicate levels of the Y-variable, from -10% (i.e., 10% population increase) at the close corner, to 100% in the far corner. Road mortality is the multiple of the best estimate (the "percent killed" values in Table 1).
